# Endometriosis: A Comprehensive Analysis of the Pathophysiology, Treatment, and Nutritional Aspects, and Its Repercussions on the Quality of Life of Patients

**DOI:** 10.3390/biomedicines12071476

**Published:** 2024-07-04

**Authors:** Gabriela Cano-Herrera, Sylvia Salmun Nehmad, Jimena Ruiz de Chávez Gascón, Amairani Méndez Vionet, Ximena A. van Tienhoven, María Fernanda Osorio Martínez, Mauricio Muleiro Alvarez, Mariana Ximena Vasco Rivero, María Fernanda López Torres, María Jimena Barroso Valverde, Isabel Noemi Torres, Alexa Cruz Olascoaga, Maria Fernanda Bautista Gonzalez, José Antonio Sarkis Nehme, Ignacio Vélez Rodríguez, Renata Murguiondo Pérez, Felipe Esparza Salazar, Ana Gabriela Sierra Bronzon, Eder Gabriel Rivera Rosas, Dante Carbajal Ocampo, Ramiro Cabrera Carranco

**Affiliations:** 1 Escuela de Ciencias de la Salud, Universidad Anáhuac Puebla, 72810 San Andrés Cholula, Mexico; gabriela.canohe@anahuac.mx; 2 Centro de Investigación en Ciencias de la Salud (CICSA), Facultad de Ciencias de la Salud, Universidad Anáhuac México Norte, 52786 Naucalpan de Juárez, Mexico; 3 Facultad de Ciencias de la Salud, Universidad Panamericana, 03920 Ciudad de México, Mexico; 4 División de Ciencias Biológicas y de la Salud, Unidad Xochimilco, Universidad Autónoma Metropolitana, 04960 Ciudad de México, Mexico; 5 Center of Excellence for Aging and Brain Repair, Department of Neurosurgery and Brain Repair, Morsani College of Medicine, University of South Florida, 12901 Bruce B. Downs Blvd., Tampa, FL 33612, USA; 6 Facultad Mexicana de Medicina, Universidad de la Salle, 14000 Ciudad de México, Mexico; 7 Departamento de Cirugía Ginecológica de Mínima Invasión, Instituto Pélvico Neurovascular, 76807 San Juan del Río, Mexico; 8 Departamento en Cirugía Pélvica, Doyenne High Quality and Multidisciplinary Treatment Center for Endometriosis, 06700 Ciudad de México, Mexico

**Keywords:** endometriosis, endometriosis nutrition, endometriosis physical activity, microbiota, chronic pelvic pain, cannabinoids in endometriosis

## Abstract

Endometriosis is a chronic proinflammatory pathology characterized by the growth of tissue similar to the endometrium outside the uterus, affecting approximately 5 to 15% of women worldwide. Suffering from endometriosis entails a complex pathophysiological process, significantly impacting the quality of life and reproductive function of affected women; therefore, it must be addressed in a personalized and comprehensive manner, as its management requires a multidisciplinary approach. This article aims to conduct a comprehensive literature review of endometriosis, not only as a pathophysiological condition but also as a significant factor impacting the social, nutritional, and mental well-being of those who experience it. Emphasis is placed on the importance of understanding and assessing the impact of the pathology to provide a better and more comprehensive approach, integrating various alternatives and strategic treatments for the factors involved in its development. The aim is to provide a complete overview of endometriosis, from its pathophysiology to its impact on the quality of life of patients, as well as a review of current treatment options, both pharmacological and alternative, in order to broaden the perspective on the pathology to improve the care of patients with this disease.

## 1. Endometriosis Generalities

Endometriosis is a chronic systemic proinflammatory condition characterized by the growth of tissue similar to the endometrium (simple columnar epithelium) outside the uterus, causing dysmenorrhea, dyspareunia, and occasionally infertility. The recruitment of proinflammatory cytokines and shifts in circulating immune cell populations secondary to the changes that come with endometriosis creates a sustained inflammation within the pelvis and involved structures [1]. The term “endometriosis” comes from the Greek “endo” meaning within, “metra” uterus, and “osis” disease; it remains a vague concept, with pelvic pain being the most common clinical symptom [2]. This condition affects 5–10% of women of reproductive age worldwide and constitutes the most common cause of pelvic pain in women [1,3]. It is a syndrome with complex etiology involving hormonal, neurological, and immunological factors [4,5].

The origin of endometrial tissue in exogenous locations is unknown and subject to controversy, with various theories attempting to explain it [4]. The most common sites where endometrial tissue is found outside the uterine cavity are superficial areas within the peritoneum; other common locations include the ovaries, fallopian tubes, uterosacral ligaments, cervix, bladder, vagina, abdominal wall, and gastrointestinal tract; less frequently, it can be found in the spleen, pleura, pericardium, or central nervous system [6].

Endometriosis poses medical, socioeconomic, and public health challenges as it is a chronic disease that significantly impacts patients’ quality of life [7,8]. It involves not only physical suffering but also a range of conditions, such as psychiatric and medical comorbidities, the need for multiple surgeries, and high treatment costs [9]. A study by Koller et al., which investigated 8276 patients with endometriosis and 194,000 controls using a logistic regression model focusing on the association of various pleiotropic loci in these patients, revealed a positive association between endometriosis and various pathologies, reporting their respective Odds Ratio (OR). It was found that endometriosis is associated with eating disorders (OR 5.02), depression (OR 4.54), and anxiety (OR 3.35) [10]. Another study by Marchandot et al. suggests an association between endometriosis and cardiovascular diseases regarding its pathophysiology and risk factors, such as atherosclerosis, hypertension, dyslipidemia, obesity, diabetes, myocardial ischemia, myocardial infarction, thromboembolic events, arrhythmias, heart failure, smoking, and environmental pollution [11]. It is important to mention that several topics are developed in this literature review, which may limit its comprehensiveness. However, we have thoroughly developed the core content of the work. 

## 2. Epidemiology

Estimates suggest that endometriosis affects between 5 and 15% of women of reproductive age worldwide [1,12,13]. The age of diagnosis typically ranges between 25 and 45 years [7]. Occasionally, it has been reported in premenarchal patients and 2–5% of postmenopausal women [5,14,15]. However, determining the actual prevalence of endometriosis is challenging and still unknown as it requires surgical visualization and subsequent histological confirmation [16]. It is estimated that 2–11% of asymptomatic women, 5–50% of infertile women, and 5–21% of women hospitalized for pelvic pain have endometriosis [17]. In adolescents, the incidence of endometriosis has been estimated at 6–10%, and it ranks as the leading cause of secondary dysmenorrhea in this population. The presence of endometriosis has been reported in 62–75% of patients with dysmenorrhea who have undergone laparoscopy and in 70% of adolescents reporting chronic pelvic pain that does not improve with NSAID or combined oral contraceptive use [18].

## 3. Pathophysiology

The development of endometriosis involves endocrine, immune, proinflammatory, and pro-angiogenic processes that interact with each other. The exact pathophysiology of endometriosis has not been conclusively established despite attempts to clarify the mechanisms through various theories and hypotheses. The existence of multiple theories creates ambiguity surrounding the disease, not only regarding its pathophysiology but also in determining both medical and surgical therapeutic approaches [4].

Among the existing theories, one of the most accepted theories relates to ectopic endometrial cells, also known as Sampson’s Theory of Retrograde Menstruation. This hypothesis establishes a flow of endometrial cells in the opposite direction through the fallopian tubes into the peritoneal cavity during the shedding of the uterine lining during menstruation [19]. The challenge with this theory is that some sites where endometriosis is found, such as ovarian and superficial peritoneal lesions, can be explained using this theory; however, other locations, such as extraperitoneal lesions or deep infiltrating endometriosis, cannot be clarified with this theory [20]. Similarly, researchers have found that endometriosis occurs in girls who have not yet experienced menarche, refuting this theory [21]. Another circumstance that helps to reject this theory is the clinical reports of endometriosis in men, which are existent despite being unusual. Finally, although researchers have estimated that up to 90% of women experience retrograde menstruation, not all develop endometriosis, implying the existence of other factors involved [22]. Additionally, the hypothesis proposed by the Group of Ivo Brosens over the past decade introduces a significant modification to Sampson’s theory. Brosens and colleagues explored the possibility that endometriosis in pre-menarcheal girls could be linked to neonatal uterine bleeding, suggesting that this early-life bleeding event serves as a precursor for the backward flow of endometrial-like cells, potentially initiating endometriosis before the onset of menstruation. While this extension of Sampson’s theory is yet to be universally accepted, its implications for understanding the very early onset of endometriosis make it a critical component of the discussion on the pathogenesis, underscoring the complexity and multifactorial nature of endometriosis. This approach not only improves the narrative on the disease’s etiology but also highlights the evolving landscape of research in endometriosis pathophysiology [23].

Likewise, another pathology characterized by the presence of endometrial glands and stroma, though of a different origin, has been described. Müllerianosis was first defined by Batt and Smith in 1989 as an organoid structure composed of Müllerian tissue remnants located in the pelvic peritoneal cavities [24]. In 2002, Redwine proposed a new theory about the origin of endometriosis based on the embryological origin of endometriosis or Mülleriosis [25]. This theory suggests that endometriosis forms during fetal development due to a defect in cell differentiation or migration of the Müllerian duct system. This could be because during embryogenesis, the coelom corresponding to the outermost layer of the mesoderm that will become the female reproductive organs can migrate outside the uterus and result in the displacement of tissues that are products of the Müllerian ducts [26]. This theory is countered by another theory called coelomic metaplasia, which suggests metaplasia is secondary to chronic inflammation due to retrograde menstrual blood and not during embryogenesis [27].

The theory that has currently gained more recognition is the Homeobox (*HOX*) genes theory. These genes are highly conserved DNA sequences that regulate the positioning of embryonic structures. *HOX* genes are involved in the development of the female reproductive system by encoding transcription factors that bind to DNA regulatory regions of target genes involved in segmental organization during embryogenesis [28]. By activating or silencing specific gene expressions, these transcription factors play a key role in directing cell differentiation and positioning in early embryogenesis. Therefore, mutations in these genes can cause genetic disorders and abnormalities in the development of the reproductive tract, leading to abnormal migration of cells involved in forming the female reproductive system [29].

## 4. Clinical

The clinical manifestations of endometriosis are multiple and diverse, depending on the location of the lesions. Endometriosis commonly develop in various site, including peritoneal superficial lesions, ovarian cysts known as endometriomas, deep endometriosis with penetration greater than 5 mm (often accompanied by fibrosis and adhesions), and extrapelvic lesions [5]. It is a systemic disease, with the main symptoms being severe dysmenorrhea, deep dyspareunia, and chronic pelvic pain, as well as intestinal symptoms, such as abdominal pain, bloating, nausea, constipation, vomiting, painful bowel movements, and diarrhea, and bladder symptoms such as hematuria and dysuria [1,30,31]. Due to the high probability of a symptom-free disease state or asymptomatic disease without evidence supported by recent diagnostic tools, endometriosis in the early or subclinical stage can be challenging to detect. Additionally, there are occasions when the intensity of the symptoms is not related to the severity of the disease [4,32]. 

Powerlessness, the need for social support, the impact on emotional well-being, and the perception of self-image are severe symptoms doctors rarely consider during consultation. Hence, a multidisciplinary approach is not usually adopted. Depression and anxiety are more common in women with endometriosis than in those without the disease; likewise, the perception of pain in these patients is altered due to central sensitization [33].

## 5. Diagnosis

Laparoscopic visualization, along with histological verification, remains the standard for diagnosing ovarian cystic endometriosis. Various classification systems exist for this pathology, with the one established by the American Society for Reproductive Medicine (ASRM) being the most widely used due to its ease of understanding by both physicians and patients [32]. Deep endometriosis can be suspected due to the predominantly intra-abdominal location of the lesions and their variation in size. It is the standard because imaging or clinical examination cannot rule out smaller, more superficial lesions [9,34,35]. The surgeon’s interest and experience determine the recognition of subtle lesions. Likewise, pathological confirmation depends on the biopsy method and the accuracy of lesion classification [9]. With a sensitivity and specificity greater than 90%, endometriomas can be reliably identified by transvaginal ultrasound or magnetic resonance imaging. With transvaginal ultrasound, a specialist can identify deep endometriosis and adhesions involving pelvic organs. Although MRI’s sensitivity to detect deep endometriosis is 94%, it is crucial to emphasize that its specificity is only 79% [36].

## 6. Treatment

When selecting the right therapy to approach endometriosis, a health professional should carefully consider each prevalent symptom and its preferences, the possible collateral effects, age, extension, disease’s location, past treatments, and economic points. In specific circumstances that involve bladder, bowel, ureters, or extrapelvic structures, as well as cases with overlapping pain conditions, it is essential to provide the patient with a multidisciplinary approach so there is adequate and integral care [37,38]. Currently, treatments include hormonal treatment and eliminating injuries, but with limited efficiency. For injuries, surgical excision with hormonal medication is the main therapeutic method; however, these methods often have side effects besides variable efficiency [5]. As seen in endometriosis, surgery usually makes an important contribution to the treatment of infertility. The condition’s stage and results must be considered while evaluating the achievement of the surgical treatment in contrast with methods of alternative management [39]. 

The options for medical therapy cover non-steroidal analgesics, hormonal contraceptives, gonadotropin-releasing hormone analogs (GnRH), and aromatase inhibitors [40]. Nevertheless, the benefit of medical management before or after the surgery is still uncertain. The omission of endometriosis before the surgery can reduce inflammation and assist in the elimination of injuries. Even though post-surgery hormonal deletion can prevent recurrence, it is essential to point out that in the different systematic reviews, neither the pre-operative nor the post-operative medical treatment has any general clinical effect [41]. It is important to mention that this review does not include surgical treatment options. 

### 6.1. Current Non-Pharmacological, Pharmacological, and Future Treatments

#### Non-Pharmacological Treatments

The treatment of endometriosis can vary according to symptoms, extent, and location of the lesions. The main approach includes surgery, pharmacotherapy, and comprehensive individual treatment. However, despite the pharmacological approach used and the effectiveness of existing therapies, alternative non-pharmacological or natural options have currently been sought to achieve a decrease in the adverse effects or complications that may arise from pharmacological treatments, thus generating a continuous search for safe and effective treatments that can be maintained in the long term, given their natural origin [42]. The pathogenesis of endometriosis involves the dysregulation of multiple physiological processes. Herbal or natural therapies aim to inhibit, interact with, and regulate cellular processes to improve proliferation, inflammation, and angiogenesis, which characterize the development of endometrial tissue outside the uterine cavity. Furthermore, as it is an estrogen-dependent pathology, the alternative therapies that have been described are primarily aimed at regulating and inhibiting the production of estrogens [43].

One of the most commonly described alternatives in the literature is the use of Resveratrol as an antiangiogenic, antioxidant, and anti-inflammatory agent. An experimental study conducted by Ricci AG et al. on murine models suggests that treatment with Resveratrol significantly reduces cell proliferation and vascular density of lesions established by endometriosis, as well as the proliferation of endometrial endothelial cells. Resveratrol is a natural phytoalexin polyphenol synthesized by plants. It has been shown to suppress cell invasion and induce apoptosis while having weak estrogenic activity. In the same study, the effect of epigallocatechin-3-gallate (EGCG) in combination with Resveratrol-induced therapy has been described as using both treatments together, leading to a reduction in lesion size with statistically significant results. However, the EGCG by itself has been shown to be effective in the treatment of benign feminine reproductive disorders because it can work as an antioxidant and target a variety of pathways, promoting apoptosis, lipid peroxidation, cholesterol abduction, and free radical scavenging, making it clinically promising as an alternative treatment [44,45]. 

On the other hand, herbal extraction with anti-inflammatory attributes exists among the described therapies. Between them, the extract of the Pueraria, also known as the kudzu flower, inhibits the aromatase activity present in endothelial cells, removing the adhesion and migration. Likewise, the hexane extract of black garlic is used for its proapoptotic, antioxidant, and anticarcinogenic activity, caused by inhibiting some signaling pathways, making it a promising alternative in preventing and treating endometriosis. Similarly, the extract of Uncaria tomentosa has been used as an alternative treatment in various inflammatory pathologies, including endometriosis, as it mediates the cell cycle by inducing apoptosis and inhibiting prostaglandin 2 (PGE2) activity [43].

Regarding curcumin, Arablou and Kolahdouz-Mohammadi have reviewed the therapeutic effects of this phenolic compound on endometriosis. They documented that it reduces the progression and invasion of endometrial cells and inflammation [46]. Moreover, Chowdhury et al., in their protocol, concluded that curcumin has beneficial effects on stromal cells in patients with endometriosis, in which a reduction in the expression of cytokines and chemokines and an inhibition of STAT3 and JNK phosphorylation in a dose-dependent manner were observed, making it a good alternative for symptoms generated by endometriosis [47]. 

Recent research about the effect of curcumin on gynecological disorders has yielded significant results. For instance, it has been demonstrated that curcumin provides significant relief of the symptoms produced in urinary infection due to its anti-inflammatory, antitumor, and antioxidant effects, in addition to its antiangiogenic properties that suggest that curcumin could be a potential alternative treatment in endometriosis acting on inflammation, oxidative stress, invasion, apoptosis, and adhesion [46,48].

Another essential investigation in animal models has demonstrated the melatonin capacity to reduce the volume of endometrial mass. In these studies, it has been observed that the cellular proliferation induced by estradiol is inhibited after 48 h of melatonin management. Additionally, it was observed how melatonin inhibited estradiol-induced epithelial–mesenchymal transition and migration of endometrial epithelial cells [46]. 

Meanwhile, the use of *Calligonum comosum* extract, in some research, has demonstrated that it is associated with the growth inhibition of endometrial injuries in development by inducing reduced vascularization, proliferation, and immune infiltration. Other alternative managements like the use of acai extract, viburnum opulus, and silimarina have been described by their antioxidants, anti-inflammatory, antiproliferative, and proapoptotic effects that meaningfully reduced the volume and proliferation of endometrial injuries [42]. 

### 6.2. Pharmacological Treatments

#### 6.2.1. Progestins

Progestins are the first line of pharmacological treatment for endometriosis. They are synthetic compounds with progestogenic activity [49]. By joining the progesterone receptors, it causes a decrease in the luteinizing hormone (LH) and follicle-stimulating hormone (FSH), which results in anovulation and amenorrhea. Additionally, they exert a hypoestrogenic effect that inhibits the inflammatory response and angiogenesis and promotes apoptosis in endometrial cells, thus blocking endometriosis and preventing dysmenorrhea. The principal use is based on eliminating or reducing the associated pain [49,50].

The versatility in the administration of these drugs makes them really helpful because they can be administered through oral, intramuscular, subcutaneous, and intrauterine routes. The most common side effects include bleeding or irregular uterine spotting, weight gain, humor swings, and bone loss (only long treatments with medroxyprogesterone acetate) [51]. 

In the treatment of endometriosis, the most commonly utilized progestins are as follows. 

#### 6.2.2. Dienogest (DNG)

Derivatives of 10-nortestosterone are progestogens administered orally and have a high specificity for the progesterone receptor. DNG has been demonstrated to essentially improve the symptoms of endometriosis, especially pelvic pain [49,52]. Furthermore, its efficacy in the size reduction in ovarian endometriomas has been observed in some patients, without affecting the ovarian reserve in the long term [53]. The side effects, such as abnormal uterine bleeding and head pain, tend to decrease the time of the treatment. Also, patients who are treated with DNG have experienced an improvement in their sexual activity and life quality [51,54]. 

#### 6.2.3. Norethindrone Acetate (NETA)

Derivatives of 19-nortestosterone are administered orally. In low doses, the NETA reduces dysmenorrhea, dyspareunia, and dyschezia, but it may cause side effects related to its androgenic activity, such as weight gain, acne, and seborrhea. Even though it offers benefits to control pain, DNG has better tolerance in most patients, which changes the progestin of election in comparison with the NETA [51,55]. 

#### 6.2.4. Medroxyprogesterone Acetate (MPA)

Derivatives of progesterone 17-OH are administered orally or by a depot formulation every three months intramuscularly or subcutaneously [51]. The MPA reduces pain associated with endometriosis and improves life quality and productivity. Nevertheless, it is related to a significant side effect: the loss of mineral bone density and an increased fracture risk. For this reason, the FDA recommends its use only when other therapies are inadequate and for a maximum period of 2 years [51,56]. 

### 6.3. Gonadotropin-Releasing Hormone Analogs

The gonadotropin-releasing hormone analogs (GnRH-a) are peptides obtained during the replacement of a D-aminoacid for the L-aminoacid native in the six positions of the native peptides of GnRH. This modification takes the resistance of the degradation for endopeptidases, which extends the occupation in the GnRH receptor. As a result, this mechanism stimulates the pituitary gland to produce LH and FSH, which increases estrogen levels; this effect is named the “sprout effect” and usually appears in the first 10 days of treatment [51,57]. This prolonged LH, FSH, and estrogen stimulation leads to a negative regulation of the GnRH-a receptors, which ultimately causes a decrease in LH, FSH, and estrogen levels. This action mechanism results in a state of hypoestrogenism that activates amenorrhea and regression of endometrial lesions. Some side effects associated with hypoestrogenism involve sleep disturbances, urogenital atrophy, and accelerated bone loss [53,58]. Despite their efficacy, GnRH-a are considered a second-line treatment due to its high cost [53]. 

### 6.4. Oral Gonadotropin-Releasing Hormone (GnRH) Antagonist

These drugs suppress the production of gonadotropin pituitary hormone while computing directly with GnRH receptors [51]. This induces a hypoestrogenic state without causing a sprout effect and shows a quicker therapeutic effect instead of GnRH-a. They are mainly administered orally and present side effects similar to the ones from GnRH-a, linked to the patients’ hypoestrogenic state. The drug of choice from this group is Elagolix, which is a new gonadotropin-releasing hormone inhibitor (GnRH); it does not have a peptide origin. It is composed of uracil-phenylethylamine and butyric acid [59]. It was approved by the FDA in 2018 and is used for the management of moderate to severe pain; it also decreases dysmenorrhea caused by endometriosis [60,61]. This treatment can be considered as a first therapeutic option for women who have a history of experimenting with side effects due to the use of contraceptive agents. Particularly, its administration is not recommended in women who have not appropriately responded to GnRH agonists or antagonists [62]. 

### 6.5. Analgesics

Although they are not a treatment for endometriosis on their own, pain relievers are utilized as first-line treatment. Nonsteroidal anti-inflammatory drugs (NSAIDs), acetaminophen, and some opioids are used to relieve pain associated with endometriosis [53]. NSAIDs are considered the first-line symptomatic treatment due to their ability to inhibit prostaglandin production and inflammation. However, it is recommended to limit their long-term use due to their side effects, such as gastric ulcers and acute kidney injury [56]. Likewise, they also decrease the stimulation of afferent nerves and directly affect nociceptive receptors to prevent the production and release of substances that generate pain [59].

### 6.6. New Treatments

Most studies that continue to be evaluated for the medical treatment of endometriosis have described that the main treatment choice is GnRH antagonists due to their effectiveness and safety [61].

A study published by Volpini et al. mentions the use of nanotechnology through emerging theranostic applications of nano products and combining phototherapy and other forms of nanotechnology as new treatment modalities for endometriosis [63]. Among the main methods that have been studied within the field of nanotechnology is the use of magnetic hyperthermia, which consists of the injection of nanoparticles for the elimination of endometrial tissue; but so far, it has only been tested in murine models, although it is not ruled out that in the future it could be a great alternative treatment [64] (Table 1). 

Light-induced therapy includes photodynamic therapy and photo thermal therapy, based on the conversation of near-infrared light into heat to induce hyperthermia. This local hyperthermia increases protein denaturation and membrane disruption, leading to cell death and reducing endometriosis. However, its limitations lie in inadequate tissue penetration and variability in the light required for the desired effect [65]. 

## 7. Nutrition Related to Endometriosis

In view of the chronic nature of endometriosis and its variability in response to different treatments, various strategies are necessary for managing these patients. One of these alternative practices is dietary control; approximately half of the women with dietary support have reported great improvement in their condition [65].

### 7.1. High Fiber, Low in Trans Fatty Acids Diet, and Its Response in Endometriosis

Estrogens play a relevant role in the pathogenesis of endometriosis, so any factor in the diet that modulates estrogen activity will be important. Increasing dietary fiber and decreasing saturated fat have been found to help reduce circulating estrogen concentrations by 10 to 25% [66].

A high consumption of trans fatty acids has been shown to increase proinflammatory markers such as Interleukin 6 (IL-6) and Tumor Necrosis Factor Alpha (TNF-α), involved in the pathogenesis of endometriosis [67].

Some fatty acids, such as Omega-6, are precursors of proinflammatory prostaglandins associated with increased pelvic pain in endometriosis [68]. However, some dietary fats have protective roles against the condition. A study conducted by Missmer et al. on premenopausal women reported fewer diagnoses of endometriosis in women with high Omega-3 consumption [69].

### 7.2. Relationship between the Consumption of Fruits and Vegetables in the Development of Endometriosis

Fruits contain high levels of antioxidants, which can reduce free radicals, oxidative stress, and, in turn, inflammation. These anti-inflammatory properties may decrease the risk of developing endometriosis [70]. A cohort study conducted by Harris et al. found that consuming high amounts of fruit, specifically citrus, reduces the risk of endometriosis by 22%. In contrast, consuming vegetables and peas/beans was associated with an increased risk of endometriosis [71].

### 7.3. Relationship between Meat Consumption and the Development of Endometriosis

Red meat consumption can be associated with high levels of estradiol, estrone sulfate, and proinflammatory markers that lead to increased inflammation and the development of endometriosis [66]. A study conducted by Yamamoto et al. found that women with a high consumption of red meat (more than two servings per day) had a 56% increased risk of endometriosis [72].

### 7.4. Vitamin Supplementation. D, C, and E and Their Help with Endometriosis Symptoms

Low levels of vitamin D (VD) have been associated with a higher risk of endometriosis and an increase in the severity of symptoms. In a clinical trial conducted by Mehdizadehkashi et al. on women with endometriosis, VD administration improved symptoms by reducing pelvic pain [73]. This may be due to VD’s anti-inflammatory effect by decreasing C-reactive protein (CRP) [67]. Vitamin C (VC) and Vitamin E (VE), being antioxidants, can help mitigate the role of inflammation in endometriosis. Supplementation with VC and VE has been shown to reduce pelvic pain, dyspareunia, dysmenorrhea, and the risk of endometriosis [66,68,74].

### 7.5. Caffeine and Endometriosis

Caffeine consumption has been shown to increase estrogen and estrone in the follicular phase, which may increase the risk of estrogen-dependent pathologies such as endometriosis [70]. In a meta-analysis conducted by Kechagias et al., no association was found between caffeine consumption and an increased risk of endometriosis. However, they did find an association between the amount of caffeine consumed and endometriosis. High amounts of caffeine (>300 mg/day) may be associated with an increased risk of endometriosis [75] (Table 2).

## 8. Microbiota and Dysbiosis

The microbiome is the conglomerate of microorganisms, their genes, and their metabolites found in a particular body structure, such as the lung or the cervicovaginal mucosa. In contrast, the microbiota refers to a community of microorganisms (predominantly bacteria, viruses, fungi, archaea, and protozoa) in a specific ecological niche [76]. A healthy gut microbiome plays a crucial role in maintaining homeostasis through mechanisms such as nutrient absorption, gastrointestinal tract maintenance, pathogen protection, and regulation of endocrine and immune processes, which collectively support overall individual health and mucosal integrity [77,78]. On the contrary, dysbiosis refers to any alteration or change that disrupts the microbiota or its composition, which could be related to disease [79,80,81].

Although there is no consensus establishing the presence of microbiota at the uterine level, this possibility has been addressed in various studies [82]. In a study conducted by Moreno et al., the presence of microbiota was found in samples of endometrial fluid, which differed from the microbiota present in vaginal aspirate samples. The endometrial microbiota has been subdivided into *Lactobacillus dominant* (more than 90% *Lactobacillus* spp.) and non-*Lactobacillus dominant* (less than 90% *Lactobacillus* spp. with more than 10% of other bacteria); it was suggested that the latter could induce a more significant inflammatory response in the endometrium [83]. On the other hand, a study by Baker et al. showed that the bacteria present in the uterus are not resident, and instead, originate from the vaginal epithelium. Bacteria can ascend from the vagina to the uterus through uterine contractions, hematogenous dissemination, and disruption of protective barriers such as cervical mucus and cervical plug [84]. 

Dysbiosis is related to abnormal estrogen metabolism [85]. Specifically, the intestinal microbiota will secrete two specific enzymes, β-glucosidase and β-glucuronidase, which act to deconjugate estrogens and increase the reabsorption of free estrogen, resulting in a higher blood concentration of estrogen levels [86,87]. Another way dysbiosis can cause endometriosis is by its ability to alter the immune system and, thus, induce inflammation by elevating proinflammatory cytokines [82]. If this inflammatory process becomes chronic and there is a higher concentration of estrogens, an ideal environment is created for the onset and progression of endometriosis [82,85]. 

Genus *Lactobacillus* is the most important bacteria in the cervicovaginal microbiome because its presence is associated with adequate gynecological and reproductive health [88]. It is also responsible for creating a hostile environment for pathogens and hostile bacteria through various mechanisms of action, such as the production of antimicrobial peptides and anti-inflammatory cytokines that help protect the epithelial cell barrier [84,89,90]. In bacterial vaginosis, the most common vaginal infection in women of childbearing age, a decrease in *Lactobacillus* along with an increase in anaerobic bacteria, is well described, which will alter the function of the epithelial cell barrier and increase the levels of proinflammatory cytokines in the cervicovaginal epithelium [90,91]. This high inflammatory response confers an increased risk for endometriosis [92]. 

## 9. Cannabis

The endocannabinoid system (ECS) was discovered in 1992 and described as a group of endocannabinoid receptors, enzymes, and ligands mainly located in the central and peripheral nervous systems. Three main elements integrate this signaling complex: (1) G protein-coupled cannabinoid receptors (CB1 and CB2); (2) endocannabinoids (endogenously produced cannabinoids), anandamide (AEA), the first isolated endocannabinoid produced by the action of N-acetyltransferase, and specific phospholipase D of N-acyl phosphatidylethanolamine (NAPE-PLD) on the phospholipid membrane and 2-arachidonoyl glycerol (2-AG) synthesized by the enzyme 1,2-diacylglycerol lipase α/β (DAGL), its production stimulated by an increase in intracellular calcium concentration; and (3) transient receptor potential vanilloid 1 (TRPV1) ion channels [93,94]. The ECS is vital in modulating various physiological processes, such as appetite, mood, memory, and pain, making it a therapeutic target for pathologies associated with chronic pain, such as endometriosis. Both endocannabinoids and cannabinoids activate CB1 and CB2 receptors located in the presynaptic region in the central and peripheral nervous systems, which can inhibit nociceptive processing and produce analgesia [94,95,96,97].

CB1 receptors are primarily synthesized in the uterus, while CB2 receptors are predominantly expressed in lymphoid tissue. These receptors are bound to inhibitory G proteins, recruiting beta-arrestins to influence arrestin-dependent signaling pathways. Oocytes express CB1 and CB2 receptors, which change location during maturation [98,99]. Endometriosis has been described as an “endocannabinoid deficiency” characterized by the ectopic growth of uterine endometrial tissue with clinical manifestations such as severe pain, dysmenorrhea, and dyspareunia [93,100]. Women with endometriosis have reported fluctuating plasma levels of endocannabinoids such as AEA, 2-AG, N-oleoylethanolamide (OEA), and N-palmitoylethanolamide (PEA). When endocannabinoid levels were associated with the severity of endometriosis pain, a correlation was found between increased plasma endocannabinoid ligands and decreased local CB1 receptor expression in individuals with endometriosis. High levels of AEA were associated with moderate to severe dysmenorrhea, while elevated levels of PEA were linked to moderate to severe dyspareunia [100]. This suggests that the dysregulation of the ECS alters the signaling of the pain response associated with endometriosis [94]. 

Bilgic et al. found that CB1 and CB2 receptors are present in lower quantities in the endometrial tissue cells of women with endometriosis compared to women without the disease [101]. Resuehr et al. also found reduced CB1 in women with endometriosis [102]. ACPA and CB65 are selective agonists for CB1 and CB2, respectively; they trigger apoptosis in endometrial glandular cells (Ishikawa cells) and cells of the endometriosis cyst wall CRL-7566 [101]. Leconte et al. demonstrated the antiproliferative effect of the non-selective agonist CB1/CB2 WIN 55212-2 on deep infiltrating endometrial stroma cells. This effect occurred through the inactivation of the Akt pathway mediated by WIN 55212-2 [103].

Sinclair et al. conducted an anonymous cross-sectional survey online via social media through endometriosis advocacy groups, asking whether cannabis use improved symptoms in patients diagnosed with endometriosis. They concluded that survey participants prefer to continue using cannabis for their condition as it alleviates pain compared to current treatments, making cannabis a potential therapeutic option to improve pain in endometriosis [104]. 

Treatments for endometriosis are ineffective, leading to frustration among women who seek different methods to alleviate their pain, including the use of cannabis. Its medicinal use has been reported since 2700 B.C. in China, as it has been shown to have anti-inflammatory and analgesic effects. In a study conducted by Armour et al., it was reported that cannabis users experienced an improvement in their endometriosis-related symptoms. However, only a small portion of the study population used this substance, indicating that more clinical studies are required to investigate its potential analgesic effect on endometriosis [105].

In contrast, studies conducted by Escudero et al. demonstrated that repeated use (2 mg/kg, once daily for 28 days) of Δ9-tetrahydrocannabinol (THC), which is the primary psychoactive component of the cannabis sativa plant, provides sustained relief of caudal mechanical hypersensitivity associated with the presence of endometriosis throughout the treatment stage, reducing painful symptoms. It is important to note that THC also inhibited the growth of ectopic endometrium without apparent consequences on eutopic endometrium and ovarian tissues. However, the main issue with the use of cannabis as an alternative treatment is the potential for numerous side effects, as well as the risk of patient abuse [106].

## 10. Physical Activity

Physical activity (PA) is considered an integral part of endometriosis treatment. For over three decades, research has been conducted on how PA influences this condition [107]. For PA to provide health benefits, it is essential to consider skeletal muscle as an endocrine organ. During muscle contraction, substances known as myokines are released, which have local effects on the muscle and distal organs such as the liver, pancreas, or adipose tissue. Additionally, exercise increases the production of leukocytes, cortisol, and adrenaline, which possess anti-inflammatory effects [108]. 

Given that endometriosis is an estrogen-dependent condition and PA can increase Sex Hormone-Binding Globulin (SHBG), it is postulated that PA may reduce bioavailable estrogens. This glycoprotein binds to estrogens with high affinity and specificity, suggesting that PA could be a protective factor [109]. In vivo experiments in mouse and rat models have demonstrated the effectiveness of PA as a protective factor, oxidative stress, disease development, and the size of endometriotic lesions, as well as its potent anti-inflammatory effect [110]. 

Similarly, PA has been shown to reduce insulin resistance and hyperinsulinemia, which are phenomena associated with endometriosis. Comparative studies between women who regularly participate in aerobic exercise at least three times a week as well as muscle strengthening exercise at least twice a week, and those who do not, suggest a reduction in the risk of endometriosis by 40% to 80% [111]. This is due to regular exercise that increases systemic levels of anti-inflammatory cytokines, which could benefit endometriosis [108,112]. 

Pain is one of the predominant aspects that profoundly affects the daily life of women with endometriosis. According to the Endometriosis Health Profile questionnaire (EHP-30), it has been observed that hatha yoga improves well-being and self-image in these women [108]. Additionally, another study conducted by Ensarit et al. suggests that regular physical activities such as walking, yoga, resistance, or muscle strength training can significantly reduce pain associated with endometriosis. The results indicate that the frequency and regularity of exercise are important factors for obtaining therapeutic benefits [113]. Some studies support the idea that regular exercise can reduce the intensity of pelvic pain associated with endometriosis. In conclusion, exercise not only affects serotonin levels and mood but can also reduce the intensity of pain associated with endometriosis. Mind–body interventions, such as yoga and meditation, may help improve women’s quality of life and overall well-being with endometriosis [108]. In a trial with a sample size of 60 women with CPP, conventional therapy was compared against conventional therapy plus yoga, where it was reported that the yoga group experienced a decrease in pain intensity and an improvement in quality of life after 8 weeks of the study [114]. 

Nevertheless, the specific effect of physical activity and exercise and the type of recommended PA have not been established [115]. Among the main reasons why the above has not been clarified are the limited number of studies conducted on this topic and the fact that in most studies, exercise in general is considered, rather than specific types being studied [116]. 

## 11. Mental Health in Endometriosis

Endometriosis is closely related to a wide range of psychiatric symptoms, particularly the presence of depression, anxiety, psychosocial stress, and a decrease in quality of life [117]. When assessing the association between endometriosis and psychological disorders, it is crucial to consider the presence of chronic pelvic pain (CPP); this is defined as non-malignant discomfort perceived in pelvic areas that persists or recurs for a minimum period of 6 months [118]. In particular, women suffering from CPP express high levels of anxiety and depression, loss of work capacity, limitations in social activities, and poor quality of life [119,120].

Sachedina and Todd examined the relationship between endometriosis and CPP in adolescents. This study highlights that endometriosis is one of the significant causes of CPP in this age group and addresses the fact that endometriosis, often not diagnosed in time, can impact the quality of life and mental health of patients [18]. 

It is essential to note that the intensity of pain does not depend on the stage of endometriosis. Therefore, women with mild endometriosis may experience severe pelvic pain, while those with more severe endometriosis may suffer less acute or chronic pain. The information above suggests the possible involvement of psychological factors affecting the pain experience in women with endometriosis [121,122]. 

In the meta-analysis conducted by Gambadauro et al., studies comparing the prevalence of women with and without endometriosis or women with endometriosis with and without pelvic pain were analyzed. The results of this study demonstrated that patients with endometriosis who had pelvic pain presented significantly higher levels of depression compared to those without pain [123]. In another study by Lorençatto et al., the prevalence of depression was compared between a group of women diagnosed with endometriosis and CPP and a group of women with endometriosis but without CPP. The results of this study showed that 86% of women with CPP had depression, while 38% of women without pain had depression. Additionally, symptoms associated with depression, such as somatic concerns, work inhibition, dissatisfaction, and sadness, were significantly higher in women with pain [124]. 

### 11.1. Anxiety and Depression

The main issues for patients with endometriosis are pain and fertility problems [125]. The symptomatology of endometriosis can compromise the mental health of patients since women with CPP face certain limitations that affect their quality of life. Romao et al. studied depression and anxiety levels in 106 patients, of whom 52 had CPP. The results showed a prevalence of anxiety of 73% in women with CPP, while the prevalence in the control group was 37%, concluding that the quality of life is lower in patients with CPP [126]. Siqueira-Campos et al. conducted a similar study investigating the prevalence and factors associated with anxiety and depression in women with and without CPP. Findings showed that the prevalence of anxiety and depression was significantly higher in women with CPP. Moreover, factors such as physical and sexual abuse were independently associated with these conditions. These results highlight the importance of systematically managing psychological factors to improve the mental health of these patients [127]. 

### 11.2. Psychological Factors and Pain Perception

It is known that chronic pain and depression commonly coexist, with 60% of patients with chronic pain also experiencing depression [128]. Centini et al. concluded that endometriosis impacts the quality of life to a greater extent than other forms of CPP [129].

## 12. Infertility, Sexual Dysfunction, Pregnancy, and Reproductive Age

### 12.1. Infertility

Endometriosis is a common condition in women of reproductive age that can decrease fertility [39]. Studies have shown reduced rates of pregnancy and live births, as well as an increase in the rate of miscarriages in women with endometriosis and adenomyosis [130]. Infertility in women with endometriosis may be due to a variety of factors, from anatomical distortions due to adhesions and fibrosis to endocrine abnormalities and immune disorders [39]. Until today, no medical or surgical treatment significantly improves fertility [131]. 

### 12.2. Sexual Dysfunction

Since endometriosis results from inflammatory reactions and infiltration of anatomical structures, this pathology can cause dysmenorrhea, dyspareunia, dyschezia, dysuria, and CPP [132]. Dyspareunia negatively impacts sexual life and social relationships. Surgical removal of endometriotic lesions leads to a reduction in dyspareunia and an improvement in sexual activity and satisfaction. On the other hand, women with endometriosis often perceive a much lower frequency of sexual intercourse compared to healthy women, and the symptoms decrease the quality of intercourse, compromising sexual activity. Sexual dysfunction is related to impairment in quality of life [132].

### 12.3. Pregnancy

In the context of pregnancy, endometriosis presents particular challenges. Although symptoms might be expected to decrease and endometriotic lesions to reduce during pregnancy, the reality is more complicated. In some cases, malignant transformation of ovarian endometriotic lesions has been observed. This situation is further complicated by factors such as chronic inflammation, adhesions, progesterone resistance, and dysfunction of genes involved in embryo implantation. As a result, pregnancy in women with endometriosis may be marked by disease-related complications such as spontaneous hemoperitoneum and intestinal complications, as well as adverse pregnancy outcomes. These include preterm birth, fetal growth restriction, hypertensive disorders, obstetric hemorrhages (such as placenta previa and placental abruption), miscarriage, or the need for cesarean section. Although current data are contradictory and do not show a strong correlation between endometriosis and these complications, except for miscarriage and cesarean section, it is evident that endometriosis can have a considerable impact on pregnancy [133]. 

### 12.4. Reproductive Age

Endometriosis is a significant condition affecting up to 5–15% of women in their reproductive age globally, highlighting its relevance in this specific age group. This stage of a woman’s life, characterized by the ability to conceive, is particularly affected by endometriosis. This disease compromises fertility and influences various aspects of health and overall well-being [1].

## 13. Conclusions

In conclusion, our comprehensive analysis of endometriosis has highlighted its multifaceted nature, encompassing various physiological, psychological, and social dimensions. Endometriosis stands as a complex disorder with far-reaching implications for the quality of life of affected individuals. The core issue is the challenge of diagnosing conditions because of the various forms they can take, some of which may show no symptoms. Even with non-invasive diagnostic methods available, laparoscopic surgery is often needed to definitively establish the diagnosis. From the epidemiological standpoint, the prevalence of endometriosis continues to present significant challenges, affecting millions worldwide and imposing substantial burdens on healthcare systems. Moreover, our exploration into the pathophysiology of endometriosis has underscored the intricate interplay of genetic predispositions, hormonal dynamics, and immune dysregulation, offering valuable insights into potential targets for therapeutic intervention. 

The treatment options for endometriosis are evolving. While conventional medications such as GnRH antagonists remain pivotal for symptom management due to their proven efficacy and safety, nanotechnology, phototherapy, and magnetic hyperthermia for targeted tissue treatment, as previously discussed, are innovative approaches that hold the potential for revolutionizing endometriosis care. Additionally, the emerging role of the microbiota presents novel opportunities for targeted therapies. Complementary approaches such as cannabis use and physical activity show promise in managing symptoms, but further research is needed to fully understand their therapeutic potential.

The endocannabinoid system (ECS) plays a crucial role in regulating pain perception, mood, memory, and appetite, emerging as a promising therapeutic target for chronic pain conditions such as endometriosis. Even though research suggests that cannabis can be a crucial element in future treatments, the use of this substance has its drawbacks like cognitive side effects and the risk of abuse. Further research is required to understand the potential long-term effectiveness of this pathway as a treatment.

As to mental health, this study highlights the intricate connection between endometriosis and psychiatric symptoms, including depression, anxiety, and psychosocial stress, underscoring the substantial impact of this condition on individuals’ overall quality of life. Chronic pelvic pain further compounds these psychological challenges, leading to compromised work productivity, social restrictions, and reduced well-being. It is essential to recognize and address these psychological dimensions to ensure comprehensive care and improve outcomes. 

In light of these findings, it is evident that a transformative shift is needed in endometriosis management, moving beyond conventional biomedical paradigms to embrace a holistic framework encompassing nutritional, psychological, and lifestyle interventions. By promoting interdisciplinary collaboration and emphasizing patient-centered care, we can enhance the quality of life and holistic well-being of individuals living with endometriosis. Nevertheless, it is critical to recognize the limitations inherent in current research and advocate for ongoing exploration into the sustained effectiveness and safety of novel treatments, alongside a nuanced understanding of the socio-cultural dynamics shaping the experiences of individuals with endometriosis. Despite the promising advances in endometriosis research in recent years, it is critical to recognize that there are still limitations inherent in current research as well as the need to advocate for ongoing exploration into the sustained effectiveness and safety of novel treatments, alongside a nuanced understanding of the socio-cultural dynamics shaping the experiences of individuals with endometriosis. In summary, our comprehensive analysis underscores the urgency of adopting a multifaceted approach to endometriosis management, one that addresses not only the physiological manifestations but also considers the psychosocial and nutritional dimensions. This integrated approach seeks to empower patients in navigating their journey with resilience and empowerment.

## Figures and Tables

**Table 1:** General principles of endometriosis treatment options: non-pharmacological and pharmacological treatments and new treatments.

**Pharmacological and Non-Pharmacological Treatment for Endometriosis**		
**Pharmacological Treatment**		
**Treatment**	**Mechanism**	**References**
Progestins		
Dienogest (DNG)	Progestin with high affinity to the receptor, which generates a hypoestrogenic effect that inhibits the inflammatory response and angiogenesis and promotes apoptosis of endometriotic cells.	[51,52,54,64]
Norethindrone acetate (NETA)	Reduces dysmenorrhea, dyspareunia, and dyschezia.	[51,55]
Medroxyprogesterone acetate (MPA)	Reduces pain associated with endometriosis.	[51,56]
Gonadotropin-releasing hormone analogs (GnRH-a).		
Elagolix	Management of moderate to severe pain and decreases dysmenorrhea.	[60,61]
**Non-pharmacological treatment.**		
**Treatment**	**Mechanism**	**Reference**
Resveratrol	It reduces cell proliferation and vascular density of lesions established by endometriosis, as well as the proliferation of endothelial cells of the endometrium.	[44,45]
Black garlic hexanic extract	It has proapoptotic, antioxidant, and anticancer activity, given by the inhibition of some signaling pathways.	[43]
Curcumin	It has a reducing effect on the progression and invasion of endometrial cells and on inflammation by reducing the expression of cytokines and chemokines.	[46,47,48]
Melatonin	Useful to reduce the volume of endometrial mass. It inhibits estradiol-induced cell proliferation, as well as epithelial–mesenchymal transition and migration of endometrial epithelial cells.	[46]
*Calligonum comosum* extract	Inhibits the growth of endometrial lesions.	[43]
**New treatments.**		
**Treatment**	**Mechanism**	**Reference**
Nanotechnology	Application of nanoproducts such as magnetic hyperthermia and phototherapy combination.	[63,64]

**Table 2:** Nutritional approach in endometriosis.

Nutritional Approach in Endometriosis		
Treatment	Mechanism	References
Increase fiber consumption	Reduces circulating estrogen concentrations by 10 to 25%.	[66]
Reside Omega-6 consumption	Fatty acids, such as omega 6, have been shown to be precursors of proinflammatory cytokines that increase pelvic pain.	[68]
Increase Omega-3 consumption	Reduces the risk of suffering from endometriosis in premenopausal women.	[69]
Increase fruit consumption	The fruit contains a wide variety of antioxidants that reduce inflammatory processes.	[70]
Reduce the consumption of red meat	Meat consumption has been associated with high levels of estradiol, estrone sulfate, and proinflammatory markers.	[66]
Maintain good vitamin D levels	Low levels of vitamin D have been associated with a higher risk of suffering from endometriosis with severe symptoms.	[73]
Reducing caffeine consumption	Caffeine consumption increases estrogen levels, predisposing to endometriosis.	[70]
